# Development and validation of a framework for the assessment of school curricula on the presence of evolutionary concepts (FACE)

**DOI:** 10.1186/s12052-021-00142-2

**Published:** 2021-02-26

**Authors:** Xana Sá-Pinto, Giulia Realdon, Gregor Torkar, Bruno Sousa, Martha Georgiou, Alex Jeffries, Konstantinos Korfiatis, Silvia Paolucci, Patrícia Pessoa, Joana Rocha, Panagiotis K. Stasinakis, Bento Cavadas, Angelica Crottini, Tanja Gnidovec, Teresa Nogueira, Penelope Papadopoulou, Costanza Piccoli, Johan Barstad, Heloise D. Dufour, Milena Pejchinovska, Alma Pobric, Dragana Cvetković, Evangelia Mavrikaki

**Affiliations:** 1 Research Centre on Didactics and Technology in the Education of Trainers, Department of Education and Psychology, University of Aveiro, Aveiro, Portugal; 2 UNICAMearth Group, Geology Section, University of Camerino, Camerino, Italy; 3 University of Ljubljana Faculty of Education, Kardeljeva ploščad 16, 1000 Ljubljana, Slovenia; 4 Alpoente, Agrupamento de Escolas de Albufeira Poente, Albufeira, Portugal; 5 Department of Biology, National and Kapodistrian University of Athens, Athens, Greece; 6 Milner Centre for Evolution, Department of Biology and Biochemistry, University of Bath, Bath, UK; 7 Department of Education, University of Cyprus, Nicosia, Cyprus; 8 Laboratorio di Scienze Sperimentali, Foligno, Italy; 9 University of Trás-Os-Montes E Alto Douro, Vila Real, Portugal; 10 Ampelokipoi Laboratory Centre for Natural Sciences (EKFE), Athens, Greece; 11 IPsantarém, Polytechnic Institute of Santarém-School of Education, Santarém, Portugal; 12 CeiED, Lusófona University, Lisboa, Portugal; 13 CIBIO/InBio InBIO, Universidade Do Porto, Centro de Investigação Em Biodiversidade E Recursos Genéticos, Campus Agrario de Vairão, 4485-661 Vairão, Portugal; 14 INIAV, National Institute for Agrarian and Veterinary Research, Oeiras and Vairão, Portugal; 15 cE3c, Centre for Ecology, Evolution and Environmental Changes, University of Lisbon, Lisbon, Portugal; 16 Department of Early Childhood Education, University of Western Macedonia, Kozani, Greece; 17 University College of Green Development, Bryne, Norway; 18 Le Cercle FSER, Paris, France; 19 Faculty of Education, “St. Kliment Ohridski” University, Bitola, North Macedonia; 20 Faculty of Science, University of Sarajevo, Sarajevo, Bosnia and Herzegovina; 21 Chair of Genetics and Evolution, Faculty of Biology, University of Belgrade, Belgrade, Serbia; 22 Faculty of Primary Education, National and Kapodistrian University of Athens, Navarinou 13A, 10680 Athens, Greece

**Keywords:** Curricula analysis, Evolution education, Content analysis, Learning goals

## Abstract

Evolution is a key concept of biology, fundamental to understand the world and address important societal problems, but research studies show that it is still not widely understood and accepted. Several factors are known to influence evolution acceptance and understanding, but little information is available regarding the impacts of the curriculum on these aspects. Very few curricula have been examined to assess the coverage of biological evolution. The available studies do not allow comparative analyses, due to the different methodologies employed by the authors. However, such an analysis would be useful for research purposes and for the development of appropriate educational policies to address the problem of a lack of evolution acceptance in some countries. In this paper we describe the steps through which we developed a valid and reliable instrument for curricula analysis known as FACE: “Framework to Assess the Coverage of biological Evolution by school curricula.” This framework was developed based on the “Understanding Evolution Conceptual Framework” (UECF). After an initial pilot study, our framework was reformulated based on identified issues and experts’ opinions. To generate validity and reliability evidence in support of the framework, it was applied to four European countries’ curricula. For each country, a team of a minimum of two national and two foreign coders worked independently to assess the curriculum using this framework for content analysis. Reliability evidence was estimated using Krippendorf's alpha and resulted in appropriate values for coding the examined curricula. Some issues that coders faced during the analysis were discussed and, to ensure better reliability for future researchers, additional guidelines and one extra category were included in the framework. The final version of the framework includes six categories and 34 subcategories. FACE is a useful tool for the analysis and the comparison of curricula and school textbooks regarding the coverage of evolution, and such results can guide curricula development.

## Introduction

Science education should contribute to increase students' scientific literacy and improve the capacity of understanding science and the processes of producing this knowledge, to ensure more citizens can apply these concepts in their daily lives and participate in scientific debates and discussions (USA National Research Council [NRC], 2007 and 2012). School curricula should be aligned with this goal. The school curriculum represents “the expression of educational ideas in practice” (Prideaux 2003, p.326). Therefore, the learning goals that a country wants its students to achieve and the skills it wants them to develop are expressed and included in its school curriculum. Different countries consider different goals and skills to be more important than others, and for this reason it is expected that curricula would vary in both type and structure (Scholl 2012). There is much discussion in the literature concerning the definition of a school curriculum (Young 2014; Bybee 2003) and its role in education (Burrill et al. 2015). If a scientific theory needs to be widely taught, understood, and accepted by the students and future citizens of a country, it should be included in the national curriculum. Curriculum may be considered a set of official policy documents delivered to teachers and typically created by the relevant ministry of education and/or other state authorities (formal curriculum) (Sanders and Makotsa, 2016). These include all the necessary topics that a teacher should teach and some guidelines on how to do so. If a scientific theory and all its associated concepts are not included in the curriculum, then students may not have the chance to learn about it at school.

Evolution is universally acknowledged as one of the most important scientific concepts and as the unifying theme in biology. Since numerous broad themes in the field of biology are threaded and held together by the theory of biological evolution, several researchers argue that understanding this theory is necessary for scientific literacy (Fowler and Zeidler 2016). Indicative of this relevance, the U.S. National Academy of Sciences (NAS) states that “few other ideas in science have had such a far-reaching impact on our thinking about ourselves and how we relate to the world” (NAS, 1998, p.21) and that “the teaching of evolution should be an integral part of science instruction” (NAS, 1999, p.2). According to the USA NRC (NRC, 2012) evolution should be considered one of the four key concepts in biology to be explored from kindergarten onward, with increasing complexity. This is supported by several researchers that emphasize the need to develop learning progressions for teaching evolution, which should be evident from the curricula and textbooks from primary education and across biology topics (e.g., Prinou et al. 2011; Vaughn and Robbins 2017).

In fact, the study of evolution promotes inter and intradisciplinary links, allowing students to interrelate concepts from biological, physical, and Earth and space sciences and use them to achieve a better understanding of the world around them, as well as to address new problematic situations (NRC 2012). Evolution is related with several daily life experiences—from explaining biodiversity, including the ecosystems inside our species, to drug resistance by bacteria, fleas or mosquitos—and a basic understanding of evolutionary processes is fundamental to address a number of key societal problems such as biodiversity loss, climate change, health or food security (Carroll et al. 2014), resistance to antibiotics and biocides and pandemics (Lederberg 1988). In addition, there is a connection between understanding evolution and negotiating societal problems. Sadler (2005), for example, found that, while examining the informal reasoning of biology majors on scenarios based on genetic engineering socio-scientific issues, their understanding of evolution strongly influenced their decision-making. Furthermore, a deeper engagement with evolution and its understanding can develop a greater knowledge of scientific and evidence-based thinking (Heddy and Nadelson 2012) and it also provides an effective context for developing a deep understanding of the Nature of Science (NoS) (Nelson, et al. 2019), which is important for promoting science literacy (Holbrook and Rannikmae 2007).

Despite its central importance in understanding biological systems and addressing some individuals’ daily life and social problems, evolution is still not well understood (or even accepted) by a large part of society, a pattern that is observed across different developmental stages, countries, cultural and religious backgrounds (Alters and Nelson 2002; Asghar et al. 2007; Athanasiou et al. 2012; Athanasiou and Mavrikaki 2013; Athanasiou and Papadopoulou 2012; Blackwell et al. 2003; Ehrlinger et al. 2008; Kruger and Mueller 2002; Miller et al. 2006, Nehm and Reilly 2007; Nehm et al. 2009a, b; Prinou et al. 2008 and 2011; Sieckel and Friedrichsen 2013; To et al. 2017; van Dijk and Reydon 2010). There are several explanations for this persistent and cross-cultural lack of evolutionary understanding including, among others:

- The presence of “cognitive bias” that lead to evolution misconceptions (Gelman 2003; Shtulman 2006; Evans 2008; Sinatra et al 2008; Kelemen 1999; Kelemen et al. 2013; Kelemen 2012; Rottman et al. 2017)
- The fact that evolutionary science integrates knowledge, norms and methods from distinct disciplines such as geology, archaeology, and subdisciplines within biology such as genetics and ecology among others (Gould 2002)
- The difference of meanings between common and scientific language, such as “adapt”, “adaptation”, “pressure” and “fitness”, among other words, that further strengthens misconceptions (Alters and Nelson 2002; Hull 1995; Rector et al., 2013)
- The perceived conflict between evolution and religious, political and personal believes (Asghar et al. 2007; Boujaoude et al. 2011; Chuang 2003; Griffith and Brem 2004; Goldston and Kyzer 2000)
- Teachers’ lack of preparedness to teach about this subject (Prinou et al. 2011; Yates and Marek 2014; Venetis and Mavrikaki 2017; Betz et al. 2019; Gresch and Martens 2019; but see Plutzer et al., 2020 for encouraging results)

The ways that educational resources, such as textbooks and school curricula, are produced may have further contributed to this pattern. In fact, in many textbooks, references to evolution and evolutionary concepts are fragmented and limited to particular chapters (Nehm et al. 2009a, b; Prinou et al. 2011) and some even reinforce common misconceptions (Prinou et al. 2011). To study the impacts of distinct countries’ curricular designs and consequent understanding of evolution by students, comparative analyses are needed. Although the acceptance and literacy about evolution has shown to vary greatly among countries (Miller et al. 2006), few studies have analysed the effect of countries’ curricula on public evolution literacy. The study of Pinxten et al. (2020) supports the hypothesis that an earlier introduction of evolution in science curricula, and a more in-depth and transversal exploration of evolutionary ideas, may help to increase both understanding and acceptance of evolution. Few curricula analyses regarding the coverage of evolutionary concepts are available in the literature, and these mostly analyse the curricula based on a general assessment of the presence or absence of the topic of evolution (Barberá et al. 1999; Tidon and Lewontin 2004), of some special topics (e.g. Quessada and Clement 2011), or the relationship between religious and scientific views (Asghar et al. 2010). However, none of these examined which major foundational and key concepts required for evolution understanding were present from the first school years onwards. Some researchers partially addressed this problem through the use of an inductive content analysis method, that is an analysis in which the coding scheme is designed based on the analysis of the curriculum and was not predefined based on a certain theoretical framework (e.g. Kuschmierz et al. 2020) or based on mixed methods that included inductive and deductive analysis (Asghar et al. 2015; Sanders and Makotsa 2016). Such research, although very helpful, lacked a framework for comparative analysis. Indeed, comparable research requires a predefined coding scheme (or framework).

Skoog and Bilica (2002) developed such a framework to analyze the science standards of the states of USA, but their focus was on a limited set of overarching evolutionary concepts and not on their foundational concepts, thus limiting their applicability to lower school grades. Some years later, Asghar et al. (2015) provided some very useful results regarding the presence of evolutionary concepts in the biology education curricula from distinct Canadian provinces and territories, basing their template of analysis on the “Understanding Evolution Conceptual Framework” (UECF). The UECF, which was developed “by a team of teachers and scientists making use of resources such as the Atlas of Science Literacy, Benchmarks of Science Literacy, and the National Science education Standards” (Scotchmoor and Thanukos 2007, pp. 232–3), is the result of a collaborative project of the University of California Museum of Paleontology and the National Center for Science education (Understanding Evolution, 2020). UECF includes the foundational as well as the advanced concepts needed to develop a sophisticated understanding of evolutionary theory (Asghar et al. 2015). It is divided into five dimensions: History of life, Evidence of evolution, Mechanisms of evolution, Nature of science, and Studying evolution. Each dimension is further developed into core ideas appropriate for each grade (K-16). Finally, each core idea is divided in subsets of related evolutionary ideas. UECF, according to its creators, is “a list of conceptual understandings regarding evolution, aligned across grade levels to help instructors identify age-appropriate learning goals for their students and understand how concepts taught at one grade level lay the groundwork for more sophisticated concepts later on” (Understanding Evolution 2020). UECF indicates which evolution concepts and mechanisms students should learn about. It is useful as an analytical framework that identifies the foundational evolutionary ideas in elementary grades, as well as specific concepts and mechanisms concerning evolution in later grades (Asghar et al. 2015). Although UECF cannot be directly used as a curricula assessment tool, it is a useful theoretical basis to inform the design of such tools. This was done by Asghar et al., who developed their own curricula assessment tool based on UECF and on the Canadian Common Framework using the concepts “related to fossils and deep time, natural selection, and human evolution” (Asghar et al. 2015, p.5). Unfortunately, this assessment tool is focused only on a limited set of evolution concepts and the study does not present much information about the assessment tool itself. This prevents other researchers from performing similar analyses. However, Asghar et al. (2015) revealed the usefulness of UECF as an initial basis for the development of a framework that could be used to analyze and compare different countries’ curricula.

In this paper we aim to develop a framework (template) supported by validity and reliability evidence that could be used to perform comparative analyses of countries’ curricula.

### Framework development methods

To develop a framework to perform comparative curricula analysis, we started by identifying scientific studies that analysed curricula for their coverage of evolution. A non-systematic search allowed us to identify the studies of Skoog and Bilica (2002) and Asghar et al. (2015). To identify additional studies performing curricula analysis regarding the coverage of evolution we have made two searches in the Web of Science: one using the “evolution” and “curriculum analysis”; a second one with “evolution” and “curricula analysis”. From these searches we did not retrieve any papers related with analysis of the curricula regarding the coverage of evolution. Given the scarcity of papers providing a methodological framework to analyse curricula regarding their coverage of evolution, we followed the example of Asghar et al. (2015) and started developing our Framework to Assess the Coverage of biological Evolution by school curricula (FACE) based on UECF.

Content analysis (Bjørnsrud and Nilsen 2011; Erdoğan, et al. 2009; Mkumbo 2009; Seker and Guney 2012) was the selected method to analyze curricula and specifically the “deductive content analysis” as this is “guided by a half-structured or structured analysis matrix” (Kyngäs and Kaakinen 2020, p.23). Based on the UECF we built a system of categories and subcategories—attributing code numbers to each category and subcategory—that we used to proceed with the content analysis. The five knowledge dimensions that UECF includes were considered as the categories for our analysis: i) History of Life, ii) Evidence of Evolution, iii) Mechanisms of Evolution, iv) Nature of Science (NoS) and v) Studying Evolution. The main learning goals that, according to UECF, support learning in these five categories were considered as subcategories. Several studies support the importance of these five categories and their subcategories as we describe below.

### History of life

Exploring and understanding the History of Life allows students to: i) explore distinct temporal scales, a threshold concept that is essential for evolution understanding (Tibell and Harms 2017); ii) understand deep time, a prerequisite to understand macroevolutionary processes that has been proven to be challenging to many students and to predict students’ acceptance of evolution (Catley and Novick 2009; Cotner et al. 2010); iii) perceive the historical patterns of temporal scales of natural environmental changes and its correlation with extinction rates and compare those with present day patterns to fully understand the human impact in the environment (Wyner and DeSalle 2020). Aligned with these goals, UECF included learning goals that address distinct time scales (turned into the subcategories 1.1 to 1.5 and 1.7 see Appendix A) including deep time (subcategories 1.1, 1.3), the geological and human induced changes and its impacts on evolution (subcategories 1.3, 1.4 and 1.6) as well as the extinction process (subcategory 1.5).

### Evidence of evolution

Recent work has shown that students’ position on the relationship between evolution and creation can be affected, among other factors, by their understanding of the scientific evidence supporting evolution (Yasri and Mancy 2016). In agreement with this evidence, UECF includes several learning goals related with the evidence for evolution (category 2 that includes subcategories 2.1 to 2.6).

### Mechanisms of evolution

Understanding the processes that cause evolution are essential not only to understanding the world around us but also to be able to address current socioscientific issues (Fowler and Zeidler, 2016; Peel et al. 2019). UECF addresses the evolutionary processes in the dimension “evolutionary mechanisms” (category 3 from FACE), which includes not only learning goals that are aligned with the key and threshold concepts proposed by Tibell and Harms (2017) to understand evolution by natural selection (subcategories 3.1 to 3.3 and 3.5 to 3.12; see Appendix A) but also learning goals that specifically address other evolutionary processes such as sexual selection (subcategory 3.7) and drift (subcategory 3.8). Although sexual selection and drift are usually much less often addressed by evolution education research and educational curricula, these play a very important role in species evolution, being fundamental for the understanding of natural world and populations, for the teaching of evolution (Price et al. 2014; Sá-Pinto et al. 2017), and in the case of drift, to address problems such as biodiversity loss (Price et al. 2014).

### Studying evolution

In alignment with recommendations for science educationeducation (NRC 2012) UECF also includes learning goals for students to understand how researchers study evolution and how knowledge from evolutionary biology can be applied in daily life contexts. These learning goals are included in the dimension “Studying Evolution” which was turned into our category 4 (with subcategories 4.1, 4.2 and 4.3).

### Nature of science

Finally, UECF also addresses students’ understanding about the nature of science (NoS), which has been considered very important for effective science education, and evolution education in particular (e.g. Freeman et al. 2014; Handelsman et al. 2006; Labov et al. 2009; Singer et al. 2012; Wieman 2014). Several studies (e.g. Rudolph and Steward 1998; Lombroso et al. 2008; Sinatra et al. 2008; Scharmann 2018; Nelson et al. 2019) show a direct correlation between accepting evolution and understanding NoS. This means that if the curricula are made with the purpose of students to not only know but also to accept evolution, then paying attention to NoS gains an extra importance. This importance was recognized by the US National Academy of Sciences, and the UECF authors. NoS was turned into our category 5 (with subcategories 5.1 to 5.5 aligned to dimensions of NoS proposed in the Appendix H of NRC 2013).

This initial version of the Framework for the Assessment of school Curricula on the presence of Evolutionary concepts (pre-FACE) was initially piloted in the Italian curriculum. This curriculum was chosen because its learning goals are phrased in a complex, sometimes ambiguous wording, allowing different possible interpretations. Therefore, it would be ideal for revealing possible gaps or weaknesses of the pre-FACE as a framework for analyzing curricula.

We are aware that we analyze the “latent content” of evolution concepts in the curriculum, as “the locus of meaning is in the content but must be inferred by recognising a pattern across elements” (Potter and Levine‐Donnerstein 1999, p. 261). In our case the unit of analysis was the “meaning unit” – “the constellation of words or statements that relate to the same central meaning” (Graneheim and Lundman 2004, p. 106)–inside the learning goals expressed in a curriculum. Each learning goal expressed in the curriculum was considered as one meaning unit, although in some rare cases a learning goal could simultaneously address two different learning goals regarding evolution learning. One example is a goal that is asking students to “relate the consequences of antibiotic misuse with increased bacterial resistance”. This requires students to understand that anthropogenic environmental changes and biological evolution are linked (subcategory 1.4), but also that evolution can be directly observed (subcategory 2.2). So, a learning goal like this includes two meaning units and each meaning unit was coded separately. For reasons of text economy from now on when we refer to learning goals we are in fact referring to meaning units regarding the content analysis.

Validity evidence was gathered following the steps proposed by Potter and Levine-Donnerstein (1999) (Table 1) and suggestions were made on the “appropriateness, meaningfulness, correctness, and usefulness” of our framework according to our results (Fraenkel et al. 2012, p. 147).

**Table 1:** Steps followed to ensure validity

Steps ensuring the validity in the latent pattern content analysis*	Steps ensuring the validity in our research
Develop a coding scheme that guides coders in the analysis of content. If the scheme is faithful to the theory in its orienting coders to the focal concepts, it is regarded as a valid coding scheme	Our coding scheme was pre-FACE which was developed based on the UECF. As described above UECF covers the major evolution ideas (see Appendix A) and has also been used by Asghar et al. (2015). Therefore, using this as a basis enhances the validity of our coding scheme
Coders have to recognise patterns in the text	Coders had to recognise patterns in the curriculum = the presence of the concepts of the pre-FACE in the curriculum under examination
Assess the decisions made by coders against some standard (norm). If the codes match the standard for correct decision making, then the coding is regarded as producing valid data. We look at the pattern of agreement that shows at least 80% of the coders making the same coding. This is a high degree of agreement, and this sets a fairly consistent norm. It means that in our analysis the codes were effective in assessing what it was intended to assess (validity) and this would be a widely held judgment (reliability)	Coders (experts with diverse profiles and expertise in the field of biology and education –some are experts in evolutionary biology, science education and science communication and some are elementary/secondary biology teachers or elementary school/biology teachers’ trainers), some working independently and some not, provided the coding. The independent coding of the data ensured that all meaning units would be identified and that none was left outside, that is, all learning goals referring to evolution are included. Codes provided by the coders were compared and the interraters’ (intercoders’) agreement assessed by using Krippendorff’s alpha coefficient (Krippendorff 2011). Acceptable results mean a widely held judgment: anyone who would read the same extract of the curriculum would be led to the same results regarding which evolution concept was covered

^*^adjusted from Potter and Levine-Donnerstein (1999, p.261 and 266)

Three Italian coders analyzed the Italian curriculum and also translated its goals into English. Two non-Italian coders analyzed the translated learning goals using the pre-FACE. We chose to include international coders–besides the Italian ones–so that we would ensure that the coders would see the curriculum for the first time. Working along with the coders on the Italian curriculum to spot any inconsistencies in the framework or overlapping categories and taking under consideration the critique of Hanisch and Eirdosh (2020), we made some adjustments to the pre-FACE in order to: *i*) join some subcategories that were redundant and/or returned overlapping results; *ii)* include guidelines to clarify the conditions under which a learning goal should, or should not, be included in a given subcategory.

Our framework, at this point the “pre-FACE-2”, consisted of the same five categories as the UECF, namely i) History of Life, ii) Evidence of Evolution, iii) Mechanisms of Evolution, iv) Studying Evolution, v) Nature of Science (NoS), but resulted in having fewer subcategories than in the beginning of the analysis. Based on this framework we performed the following analysis. Each subcategory was assigned a number where the first digit identifies the main category to which an idea belongs, and the next digit(s) identifies the specific subcategory (see Table 2).

**Table 2:** Conceptual framework for the analysis of school curricula regarding evolution (pre-FACE-2*)

Category	Subcategrory
1. History of life	1.1 Life has been on Earth for a long time
	1.2 Present day life forms are related to past life forms
	1.3 Large scale environmental changes (caused by geological, geophysical, astronomical factors) and biological evolution are linked
	1.4 Anthropogenic environmental changes and biological evolution are linked
	1.5 Many life forms that once existed have gone extinct
	1.6 Rates of evolution vary
	1.7 Life forms/species/ change through time
2. Evidence for Evolution	2.1 Similarities and/or differences among existing organisms (including morphological, developmental, and molecular similarities) provide evidence for evolution
	2.2 Evolution can be directly observed
	2.3 The fossil record provides evidence for evolution
	2.4 The geographic distribution of extant species provides evidence for evolution
	2.5 Artificial selection provides evidence for evolution
	2.6 Organisms’ features, when analysed in relation to their environment provide evidence for evolution
3. Mechanisms of Evolution	3.1 Evolution is often defined as a change in allele frequencies within a population
	3.2 There is variation within a population
	3.3 Living things have offspring that inherit many traits from their parents but are not exactly identical to their parents
	3.4. Evolution occurs through multiple mechanisms
	3.5. Natural selection acts on the variation that exists in a population
	3.6 Inherited characteristics affect the likelihood of an organism’s survival and reproduction
	3.7 Sexual selection occurs when selection acts on characteristics that affect the ability of individuals to obtain mates
	3.8 Genetic drift acts on the variation that exists in a population
	3.9 Fitness is reproductive success—the number of viable offspring produced by an individual in comparison to other individuals in a population/species
	3.10 Species can be defined in many ways
	3.11 Speciation is the splitting of one ancestral lineage into two or more descendant lineages
	3.12 Evolution does not consist of progress in any particular direction
4. Studying evolution	4.1 Scientists study multiple lines of evidence about evolution
	4.2 In everyday life we can find applications of evolutionary biology
	4.3 Classification is based on evolutionary relationships
5. Nature of Science	5.1 Science is a human endeavor (achievement)
	5.2 Science provides explanations for the natural world
	5.3 Science is based on empirical evidence
	5.4 Scientific ideas can change through time
	5.5 Scientific theories are built through a transparent collective endeavor

^*^please note that the final version of FACE is presented in Table 4

To characterize a learning goal, a coder should consider at first the category in which it fits, then decide about the specific subcategory, and finally record every occurrence in the analyzed curriculum.

### Data used for the development of the framework

Within the European context exists a wide range of curricula designs and traditions. In Scandinavian countries and in the UK, school curricula are designed in a highly general form, only mentioning general topics for the schools and teachers themselves to be the responsible executors of content. For example, in the official Norwegian curriculum, evolution is barely mentioned, and officials are trusting teachers on how the formal content should be adapted and delivered to the students (Udir 2013 and 2020). It is self-evident that this kind of curricula were not suitable to be analyzed with the proposed framework. Thus, in this study we chose among a specific tradition of curriculum-development that is characterized by a more detailed prescription level; this fits many European countries but does not aim to reflect the whole range of European curricula traditions. Four European countries’ curricula of this kind (Greece, Italy, Portugal and Slovenia) were used to test the developed framework.

Given the differences between countries' school systems, we decided to analyze grades 1–9 Biology or Science curricula, or other subjects in which Biology is taught, if the latter did not exist as a separate subject in the school curriculum of a given school grade/country. An exception was made for Italy, for which we analyzed grades 1–10, as the official curriculum considers the 9th and 10th grades together. Although important evolution learning goals may be addressed in Geography, Geology, or History, the learning goals of these disciplinary fields were only analysed if they were taught in the same discipline that also addressed Biology learning goals.

In three out of four countries (i.e., Portugal, Slovenia and Greece) 9th is the grade until which all students share the same compulsory subjects and programs. After the 9th grade (after 8th grade in Italy), students are usually allowed to choose distinct educational branches, some of which do not include any biological discipline (information about the official documents analyzed and the distinct educational systems provided in Appendix B).

In many countries, although evolution is explored more in depth in higher grades, several organizations and researchers argue for the inclusion of evolutionary ideas starting in the first school years (Campos et al. 2013; Emmons et al. 2017; Kelemen et al. 2014; NRC 2012). This perspective motivated developing a framework for curriculum analysis that could be applied to lower school grades to study and guide curricula construction.

### Reliability and coding process

To perform reliability tests in content analysis (Krippendorff 2004, p. 212, 219), it is important to use “several researchers with diverse personalities”—like the authors of this paper who are characterized by various professional and educational profiles and in many cases were coders. The coders worked in differing environments (i.e. different origins of coders in our case) and demonstrated reproducibility (intercoder reliability; i.e. ‘two or more individuals, working independently of each other, applying the same recording instructions to the same units of analysis’; Krippendorff 2004, p. 219). More than one coder applied the same coding scheme to the same units of analysis; a minimum of two coders from each country (local coders) independently read the curriculum of their country and identified any evolutionary goals they could find in these documents. These coders generated a table where each learning goal would occupy a cell in a line (with very few exceptions where a learning goal could include more than one meaning unit, as explained above). In the cell right next to it they were asked to write the translation of this text in English, which was checked by the rest of the national team members to be consistent with the meaning of the initial text. This procedure allowed international coders (one or two foreign coders who had access only to the learning goals but not to the coding) to contribute a "blind" analysis. A “national coordinator” from each country gathered all coders’ results in one file (presented in Table 3) and gathered reliability evidence. After that, he/she (i) organized meetings with his/her country’s local coders to discuss results, (ii) identify cases in which many disagreements occurred, and (iii) propose possible changes to be included in the framework to address these problems. The problems and solutions found in each country were then discussed by the team of national coordinators who produced changes to the FACE.

**Table 3:** An example of the resulting file listing and coding the learning goal(s) in a country’s curriculum

Subject	Exact text in country’s language	Exact text in english	Coder1	Coder2	Coder3	Cycle/grade
Biology	*[…] riconoscere nei fossili indizi per ricostruire nel tempo le trasformazioni dell’ambiente fisico, la successione e l’evoluzione delle specie*	(the student) Recognise that fossil records are the clues to reconstruct environmental changes over time, species succession and evolution	23*	23	23	Middle school (grades 6th-8th)

^*^for the meaning of the coded numbers see Table 2

**Fig. 1:**
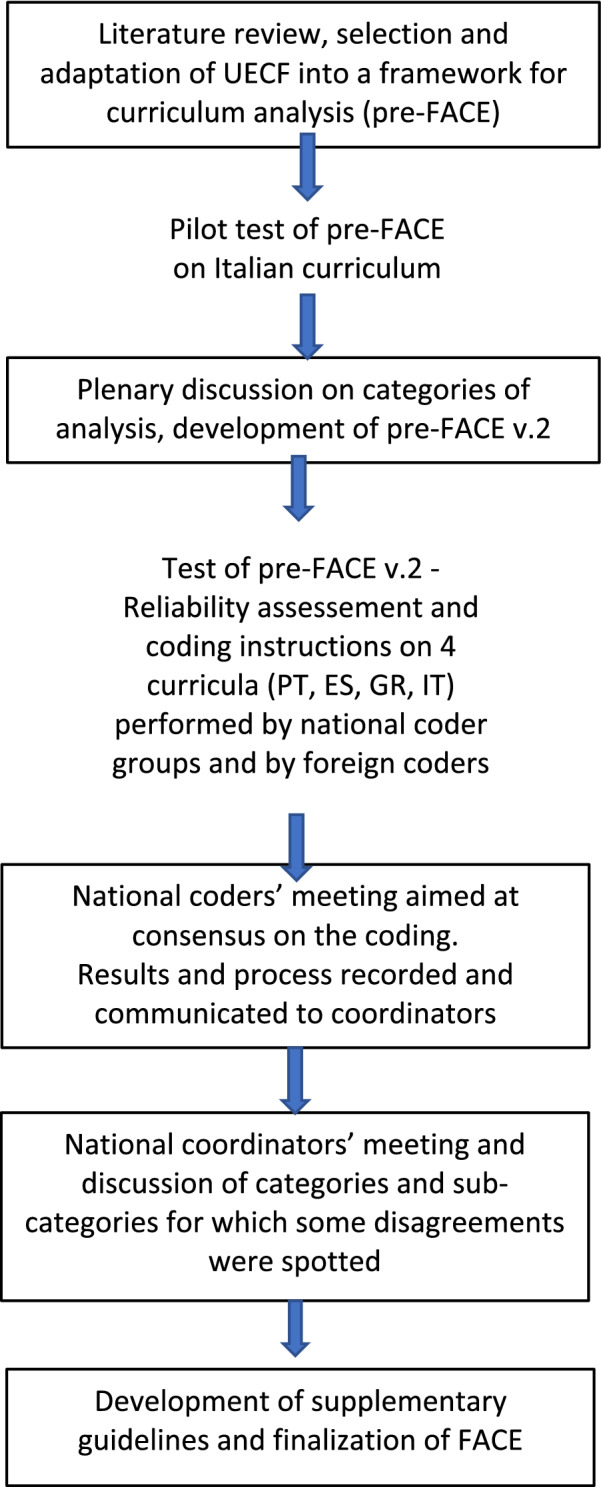
Description of the process that led to the development of FACE

The phases of FACE development are summarized in Fig. 1. The reliability of all coders for each curriculum was tested by Krippendorff’s alpha using IBM(c) SPSS 25 and the “syntax kalpha” created by Hayes and Krippendorff (2007).

## Results and discussion

Our goal was to develop a valid and reliable framework for researchers to assess school curricula according to whether they address the ideas, concepts, and mechanisms that are necessary to understand evolution. Across the four countries included in this study we found evidence supporting the presence of learning goals addressing 29 from the 33 subcategories initially included in this analysis (see Table 2 for the framework used and Table 4 for the final version of FACE). Reliability was calculated based on Krippendorff’s alpha coefficients (Table 5) and results confirmed coder reliability for each country (Krippendorff 2011).

**Table 4:** Analytical presentation of the FACE with guidelines and examples

Category	Subcategory	Guidelines	Examples
1. History of life	1.1 Life has been on Earth for a long time	Do not apply this to learning goals that do not explicitly mention time	Through billions of years of evolution, life forms have continued to diversify in a branching pattern, from single-celled ancestors to the diversity of life on Earth today. (UECF) Life on Earth 3.8 billion years ago consisted of one-celled organisms similar to present-day bacteria. (UECF) Origin of life and of first eukaryotic cells. (IT, 9th-10th grades Technical/Vocational)
	1.2 Present day life forms are related to past life forms	A learning goal considered to represent subcategory 1.2 may ask students to understand: · species’ shared ancestry · species descend from past species Do not apply to learning goals that only refer to genealogical trees of a family and relationships between individuals from one generation to the other	There is evidence of eukaryotes in the fossil record from about one billion years ago; some were the precursors of multicellular organisms. (UECF) The early evolutionary process of eukaryotes included the merging of prokaryotic cells. (UECF) To mention and explain the evidence in favor of the common origin of the living things. (GR 9th grade) To describe human evolutionary history, with special focus on the complexity of hominids’ phylogenetic tree (IT, 9th-10th grades Technical/Vocational)
	1.3 Large scale environmental changes (caused by geological, geophysical, astronomical factors) and biological evolution are linked	Do not apply to learning goals that refer to anthropogenic environmental changes	Tectonic plate movement has affected the evolution and distribution of living things. (UECF) Living things have had a major influence on the composition of the atmosphere and on the surface of the planet. (UECF) At least one mass extinction on planet Earth has been definitively linked to an asteroid impact. (UECF) Relate the influence of living beings to the evolution of the earth's atmosphere and the greenhouse effect on the earth. (PT, 8th grade) Understand the characteristics of planet Earth that allowed the emergence and evolution of life (PT, 8th grade)
	1.4 Anthropogenic environmental changes and biological evolution are linked	Do not apply to goals referring to natural environment's change	Humans directly impact biodiversity, which may then impact future evolutionary potential. (UECF) To raise their awareness of endangered animals and plants. (GR 1st grade) Recognise the changes made by human activity to habitats and the impact on the ecosystem. (GR 2nd grade) Relate the increase in world population and consumption with changes in the quality of the environment (destruction of forests, pollution, depletion of resources, extinction of species, etc.), recognising the need to adopt individual and collective measures that minimize the negative impact. (PT, 4th grade) Relate the consequences of antibiotic misuse with increased bacterial resistance. (PT, 9th grade).***
	1.5 Many life forms that once existed have gone extinct	Apply to learning goals that either explicitly or not refer to species’ extinction and not just beings	Mass Extinction can result from environmental change. (UECF) Extinctions may create opportunities for further evolution in other lineages to occur. (UECF) Identify endangered or even extinct plants and animals by investigating the reasons that led to this situation. (PT, 4th grade)
	1.6 Rates of evolution vary		Rates of speciation vary. (UECF) Evolutionary change can sometimes happen rapidly. (UECF) Some lineages remain relatively unchanged for long periods of time. (UECF)
	1.7 Life forms/species/ change through time	Do not apply to learning goals related to development	To recognise species succession and evolution. (UECF) To refer and describe the stages of evolution of the human species. (GR 9th grade)
2. Evidence for evolution	2.1 Similarities and/or differences among existing organisms (including morphological, developmental, and molecular similarities) provide evidence for evolution	A learning goal considered to represent subcategory 2.1 may ask students to recognise: · that life is diverse · differences and similarities between species · similarities and differences between species result from evolution Do not apply to learning goals that: · are related with fossils. These may be characterized by subcategory 2.3 · learning goals that are explicitly related with intraspecific diversity. For these, apply subcategory 3.2	Not all similar traits are homologous; some are the result of convergent evolution. (UECF) All life forms use the same basic DNA building blocks. (UECF) To distinguish morphological or functional characteristics related to food intake or digestion and to relate them with the evolution of organisms. (GR 7th grade) To identify similarities and differences in breathing across different categories of living organisms and identify those that are evidence of evolution. (GR 7th grade) To recognise similarities and differences in the physiological functions of different living beings. (IT 6th-8th grades) categorize living beings according to similarities and observable differences (animals, types of: coating, feeding, locomotion and reproduction; plants: root type, stem type, leaf shape, deciduous/persistent leaf, flower color, fruit and seed, etc.). (PT, 2nd grade) Characterize some of the existing biodiversity at local, regional and national level, presenting examples of relationships between flora and fauna in different habitats. (PT, 5th grade) Distinguish eukaryotic from prokaryotic cells through microscopic observations. (PT, 8th grade) Identify, name and compare different living beings and environments. (SL, 1st-2nd grade) Find differences and similarities between plants and animals. (SL,1st-2nd grade)
	2.2 Evolution can be directly observed		Relate the consequences of antibiotic misuse with increased bacterial resistance (PT, 9th grade). ***
	2.3 The fossil record provides evidence for evolution	Can also be applied to goals asking students to recognise that there are similarities and differences among fossils and living organisms	The sequence of forms in the fossil record is reflected in the sequence of the rock layers in which they are found and indicates the order in which they evolved. (UECF) Radiometric dating can often be used to determine the age of fossils. (UECF) To recognise in the fossil record the clues to reconstruct environmental changes along time. (UECF) The similarities and differences between species in the fossil record and between these and extant species provide evidence for evolution. (UECF) Fossil records are the clues to reconstruct environmental changes over time, species succession and evolution. (IT, 6th-8th grades) Explain the contribution of the study of fossils and fossilization processes to the reconstruction of the history of life on Earth. (PT, 7th grade)
	2.4 The geographic distribution of extant species provides evidence for evolution	A learning goal considered to represent subcategory 2.4 may ask students to understand that: · current geographic distribution of species often reflects how geological changes influenced lineage splitting · current distribution of species provides evidence of evolutionary processes	Insular species have closely related species in the continental areas. (UECF) Some taxonomic classes can only be found in some places. (UECF)
	2.5 Artificial selection provides evidence for evolution	A learning goal considered to represent subcategory 2.5 may ask students to understand that: · selective breeding can produce offspring with new traits · artificial selection provides a model for natural selection	People selectively breed domesticated plants and animals to produce offspring with preferred characteristics. (UECF)
	2.6 Organisms’ features, when analysed in relation to their environment provide evidence for evolution	A learning goal considered to represent subcategory 2.6 may ask students to: · recognise that plants and animals have features that allow them to live in various environments · understand that there is a fit between organisms and their environments, though not always a perfect fit · understand that an organism’s features reflect its adaptation to their environment Do not apply to learning goals that ask students to recognise the existence of similarities and differences between organisms that are not under the light of the environment. Such learning goals may be applied to subcategory 2.1	There is a fit between organisms and their environments, though not always a perfect fit. (UECF) Some traits of organisms are not adaptive (UECF) Features sometimes acquire new functions through natural selection. (UECF) Form is linked to function. (UECF) An organism’s features reflect its adaptation to their environment. (UECF) To recognise in other living organisms, with respect to their environments, similar needs as her/his own. (IT, 1st-3rd grades) Relate the characteristics of living beings (animals and plants) with their habitat. (PT, 2nd grade) Relate the characteristics (body shape, coating, organs of locomotion) of different animals to the environment in which they live. (PT, 5th grade) Identify morphological and behavioral adaptations of animals and their responses to changes in water, light and temperature. (PT, 5th grade) Relate abiotic factors—light, water, soil, temperature—with their influence on ecosystems, presenting examples of adaptations of living beings to these factors and articulating with knowledge of other disciplines (e.g. Geography). (PT, 8th grade)
3. Mechanisms of Evolution	3.1 Evolution is often defined as a change in allele frequencies within a population		
	3.2 There is variation within a population		Variation of a character within a population may be discrete or continuous. (UECF) Recognise the diversity between (…) organisms of the same species. (GR 7th grade) To observe the variability of individuals within species. (IT 6th-8th grades) Recognise similarities and differences between people. (SL, 1st grade)
	3.3 Living things have offspring that inherit many traits from their parents but are not exactly identical to their parents	A learning goal considered to represent subcategory 3.3 may ask students to understand that: · parents and offspring, as well as siblings, share features but still differ from each other; · recombination and mutations in reproductive cells result in new heritable traits and are sources of diversity; · recombination and mutations are random processes	Variation is the result of genetic recombination or mutation. (UECF) Continuous characters are generally influenced by many different genes. (UECF) Mutation is a random process. (UECF) Organisms cannot intentionally produce adaptive mutations in response to environmental influences. (UECF) Recognise mutations (not in the independent gene combination and cross-linking) as the mechanism for generating genetic diversity. (GR 9th grade) Recognise that living beings reproduce and that their offspring have characteristics similar to their parents, but also differ in some of them. (PT, 3rd grade) Explain the relationship between hereditary factors, genetic information and the way sexual reproduction conditions intraspecific diversity and population evolution. (PT, 9th grade) (…) they learn that animals have offspring that usually come from a male and female, and that their offspring are similar. (SL, 2nd grade)
	3.4. Evolution occurs through multiple mechanisms	Do not apply to learning goals that simply mention one evolutionary mechanism Learning goals that just mention natural selection or genetic drift, they should be assigned to subcategories 3.5 and 3.8 respectively	
	3.5. Natural selection acts on the variation that exists in a population	A learning goal considered to represent subcategory 3.5 may ask students to understand that natural selection can act at multiple hierarchical levels such as genes, cells, individuals, populations, species, and larger clades Do not apply to learning goals that mention more than one evolutionary process like “Natural selection and genetic drift act on the variation that exists in a population”. For these learning goals apply subcategory 3.4	Evolution results from selection acting upon genetic variation within a population. (UECF) Natural selection acts on phenotype as an expression of genotype. (UECF) The amount of genetic variation within a population may affect the likelihood of survival of the population; the less the available diversity, the less likely the population will be able to survive environmental change. (UECF) Natural selection sometimes favors heterozygotes over homozygotes at a locus. Heterozygote advantage preserves genetic variation at that locus (i.e., within the population, it maintains multiple alleles at that locus). (UECF) To define natural selection and describe the mechanism by which living organisms evolve. (GR 9th grade) Explain the need for the intervention of sex cells in the reproduction of some living beings and their importance for the evolution of the species. (PT, 5th grade)
	3.6 Inherited characteristics affect the likelihood of an organism’s survival and reproduction	A learning goal considered to represent subcategory 3.6 may ask students to understand that advantageous traits often persist in a population. Advantageous traits depend on the environment and the selective pressures it imposes	Depending upon the environment, some living things will survive better than others. (UECF) Environmental changes may affect an organism's ability to survive. (UECF) Over time, the proportion of individuals with advantageous characteristics may increase (and the proportion with disadvantageous characteristics may decrease) due to their likelihood of surviving and reproducing. (UECF) Populations, not individuals, evolve. (UECF) Traits that confer an advantage may persist in the population and are called adaptations. (UECF)
	3.7 Sexual selection occurs when selection acts on characteristics that affect the probability of obtaining a mate		Sexual selection can lead to physical and behavioral differences between the sexes (UECF)
	3.8 Genetic drift acts on the variation that exists in a population	Do not apply to learning goals that mention more than one evolutionary process like “Natural selection and genetic drift act on the variation that exists in a population”. For these learning goals apply subcategory 3.4	Smaller populations are more strongly affected by genetic drift than are larger populations. (UECF) Genetic drift can cause loss of genetic variation in a population. (UECF) Founder effects occur when a population is founded from a small number of individuals. (UECF) Bottlenecks occur when a population's size is greatly reduced. (UECF) Evolution results from genetic drift acting upon genetic variation within a population. (UECF)
	3.9 Fitness is reproductive success — the number of viable offspring produced by an individual in comparison to other individuals in a population/species		An organism's fitness depends on both its survival and its reproduction. (UECF) Fitness is often measured using proxies like mass, number of matings, and survival because it is difficult to measure reproductive success directly. (UECF)
	3.10 Species can be defined in many ways	A learning goal considered to represent subcategory 3.10 may ask students to: · provide one or more species definition · mention that hybrids can occasionally result from mating between distinct species or form	There are many definitions of species. (UECF) Some hybrids have increased fitness relative to their parents. (UECF)
	3.11 Speciation results from the splitting of one ancestral lineage into two or more descendant lineages		Speciation is often the result of geographic isolation. (UECF) Speciation requires reproductive isolation. (UECF) Occupying new environments can provide new selection pressures and new opportunities, leading to speciation. (UECF)
	3.12 Evolution does not consist of progress in any particular direction		
4. Studying evolution	4.1 Scientists study multiple lines of evidence about evolution	A learning goal considered to represent subcategory 4.1 may ask students to understand that: · scientists study living beings and how these are related to each other · our knowledge of evolution is constructed and continuously refined by multiple lines of evidence	Scientists use fossils to learn about past life. (UECF) Scientists use multiple lines of evidence (including morphological, developmental, and molecular evidence) to infer the relatedness of taxa. (UECF) Scientists use experimental evidence to study evolutionary processes. (UECF) Scientists use artificial selection as a model to learn about natural selection. (UECF) Evolutionary trees (e.g., phylogenies or cladograms) are built from multiple lines of evidence. (UECF) Explain the contribution of the study of fossils and fossilization processes to the reconstruction of the history of life on Earth. (PT, 7th grade)
	4.2 In everyday life we can find applications of evolutionary biology		To recognise, by experiencing plant cultivation and animal raising, that every organism’s life is related to other and different life forms. (IT, 4th-5th grades) The variety of living beings and the complexity of their structures and functions are subject of the study of evolution and systematics, Mendelian genetics and organism-environment interactions, with the aim of valorisation and maintenance of biodiversity. (IT, 9th-10th grades)
	4.3 Classification is based on evolutionary relationships	Do not apply to learning goals that explore classifications that are not clearly based on evolutionary relationships Apply to learning goals that explore classifications that are nested and biologically relevant (phylogenetically correct)	To classify them (organisms) according to specific criteria, to identify structural and functional similarities and differences, and relate them to the needs created by the environment in which they live. (GR general goals for 7th-9th grades) To classify typical living organisms according to classification rules. (GR 7th grade) To explain the meaning of classification and the parameters most commonly used to classify organisms. (IT 9th-10th grades Technical/Vocational)
5. Nature of Science	5.0 Understanding the NoS	Goals that mention the importance of exploring NoS that cannot be attributed to any of the subcategories below should be considered as belonging to subcategory 5.0	Value the nature of science, continuing the development of scientific methodology in its different stages (Pt, from the 1st to the 4th grade)
	5.1 Science is a human endeavor (achievement)		Recognise that science is a human activity, with its own goals, procedures and ways of thinking, through the exploration of current or historical events that document its nature. (PT,5th and 6th grades)
	5.2 Science provides explanations for the natural world		Scientists can test Ideas about events and processes long past, very distant, and not directly observable. (UECF) To use, as possible, the knowledge they achieve to interpret, phenomena, processes or problems that occur (…). (GR general biology goals 7th-9th grades)
	5.3 Science is based on empirical evidence	A learning goal considered to represent subcategory 5.3 may ask students to understand that science is based on evidence collected with our senses and extensions of our senses	Scientists use multiple research methods (experiments, observational research, comparative research, and modeling) to collect data. (UECF) Scientists use multiple research methods (experiments, observations, comparisons, and modeling) to collect evidence. (UECF)
	5.4 Scientific Ideas can change through time	A learning goal considered to represent subcategory 5.4 may ask students to understand that scientific knowledge is open to question and revision depending on new ideas and/or new evidence	
	5.5 Scientific theories are built through a transparent collective endeavor	A learning goal considered to represent subcategory 5.5 may ask students to understand that: · science always exposes ideas to testing · scientists may explore different hypotheses to explain observations · accepted scientific theories must survive rigorous testing and be supported by multiple lines of evidence to be accepted · scientific controversy and debate within the community contribute to scientific progress	Scientists test their ideas using multiple lines of evidence. (UECF)
6. Development of scientific practices	6.0 Development of practices that scientists employ as they investigate and build models and theories about the world. ****	A learning goal considered to represent subcategory 6.0 may ask for: · students’ engagement and understanding scientific methodologies and practices · students developing the scientific way of thinking	Recognise the importance of the scientific method for the study of life processes. (GR 7th grade) The student explores phenomena with a scientific approach: […] observes and describes the unfolding of events, asks questions based on personal hypotheses, proposes and realises simple experiments. (IT grades 1st-5th) Know how to ask questions, raise hypotheses, make inferences, prove results and know how to communicate, recognizing how knowledge is built. (PT, 1st-4th grade) Build scientific explanations based on scientific concepts and evidence, obtained through the performance of diversified practical activities—laboratory, experimental, field—and planned to try to answer formulated problems. (PT, 5th-6th grade) Implement practical investigations, based on systematic observation, modeling and laboratory/experimental work, to address problems related to terrestrial materials, diversity of living beings and their interactions with the environment. (PT, 5th-6th grade) Plan and implement practical investigations based on systematic observation, modeling and work laboratory/experimental, to respond to problems related to the dynamics of planet Earth and to the evidence that helps tell its story. (PT, 7th-9th grade)

^*^The examples provided are either from the UECF (Understanding Evolution Conceptual Framework) or they are goals from the analyzed curricula

^**^Guidelines came up through this analysis and are considered important to be taken under consideration for future analyses

^***^Learning goals such as this are an example of the fact that some learning goals in the curriculum may be attributed to achieving more than one subcategory

^****^This subcategory is phrased according to NRC (2012)

Although Krippendorff’s alpha value was always above the lowest acceptable level of alpha (0.67, Krippendorff 2011), several issues have been identified during the process of the development of the framework. To overcome these problems, following the suggestion of Potter and Levine-Donnerstein (1999, p. 267) to “provide formulae for weighting the different elements so that [future] coders will know how to sort through conflicting sets of cues as well as how to handle other coding problems”, we provided guidelines to be applied in specific cases. One of them concerns classifying learning goals that relate biological structure and function. In FACE, the subcategory 2.7 “Organisms’ features, when analyzed in relation to their environment provide evidence for evolution” (Table 2) was derived from the UECF which provided as an example that “Form is linked to function.” However, this subcategory sparked an intense debate in our analysis of the school curricula, mostly when trying to code learning goals that would link structure and function of internal organs without mentioning the organism’s living environment. For example, in the Portuguese “Essential Learning Goals Guidelines” it is written: “Relate the organs of the male and female reproductive system with their function” (6th grade; Portuguese Government/Ministry of Education, 2018f p10); “Identify the morphology and anatomy of the heart of a mammal, explaining its main constituents and their respective functions” (9th grade, Portuguese Government/Ministry of Education, 2018i p9); and in the Italian curricula we read: “The student can recognize in her/his organism structures and functions at macroscopic and microscopic levels” (6th-8th grades). To overcome the uncertainty of whether one should attribute subcategory 2.7 in these cases or not we decided that a learning goal fits in this subcategory only if it enables the connection of a particular feature of the organism and its external environment (see Table 4 for the final version of FACE).

Another problem identified during the application of our framework arose with subcategory 3.2—“There is variation within a population”—and subcategory 2.1—Similarities and/or differences among existing organisms (including morphological, developmental, and molecular similarities) provide evidence for evolution”—as these were sometimes misused by some of our members, who would consider cases of intraspecific variability belonging to 2.1. To solve this problem, we propose that: i) coders should assess whether the learning goal is focusing on the mechanisms of evolution or the evidence of evolution, as these two subcategories are part of different categories; and ii) the subcategory 2.1 to be applied only for learning goals that mention interspecific diversity or diversity among higher taxonomic levels (example prokaryotic versus eukaryotic cells, see Table 4 for the final version of FACE).

A similar problem was raised by the interpretation of the subcategory 1.2 “Present day life forms are related to past life forms”. When classifying learning goals related with genealogical trees, some coders applied this subcategory to relationships between individuals of the same species. To avoid this, we included a guideline stating that subcategory 1.2 should only be applied to learning goals mentioning distinct species and not distinct individuals of the same species (see Table 4 for the final version of FACE).

All coders referred to goals they identified describing the need for students to engage in scientific practices or recognize the importance of scientific methods, such as:

- “Know how to ask questions, raise hypothesis, make inferences, prove results and know how to communicate, recognizing how knowledge is built” (Portugal, Essential Learnings 1st to 4th grades; Portuguese Government/Ministry of 2018a p8; 2018b p9; 2018c p9; 2018d p10 respectively); “implement practical investigations, based on systematic observation, modeling and laboratory/experimental work, to address problems related to terrestrial materials, diversity of living beings and their interactions with the environment. (…) Build scientific explanations based on scientific concepts and evidence, obtained through the performance of diversified practical activities—laboratory, experimental, field—and planned to try to answer formulated problems.” (Portugal, Essential Learnings 5th to 6th grades; Portuguese Government/Ministry of Education, 2018e p4; 2018f p4).
- “[The student] Explores phenomena with a scientific approach”; “The pupil observes and describes the unfolding of events, asks questions based on personal hypotheses, proposes and realizes simple experiments, with the help of the teacher” (Italy, learning goals for grades 1st to 5th).
- “Is able to collect qualitative and quantitative data by observing and performing measurements, to record and present them appropriately” (Slovenia, subject Science in 6th and 7th grade).

Although engaging in scientific practices is fundamental to fostering the development of students’ scientific literacy (NRC, 2012), goals like these were not directly described in our framework of analysis. But most of the local coders recognized these and similar learning goals as belonging to the subcategories referring to Nature of Science (NoS), mainly based on the assumption that engaging in scientific practices could provide a chance to get better acquainted with the NoS. Furthermore, coding of these learning goals was among the ones with the least consensus between the coders. The confusion between scientific practices, scientific inquiry and NoS is common and longstanding (reviewed by Lederman 2019). However, research results show that students will only learn about the NoS if this is explicitly integrated in the instruction (reviewed by Lederman 2019). To further improve our framework, we recommended the introduction of a new and independent category, which would allow researchers to be more precise and include in this category the learning goals that are related with the development of scientific practices as defined by NRC (2012). Another issue related with NoS was the fact that, in some countries, some official documents mention the importance of valuing the NoS but they do not differentiate the dimensions of the NoS to be learned by students, therefore precluding its assignment to any subcategory of NoS present in our framework. One example of such statements can be found in Portuguese official documents from 1st to 4th grade that mention in its introductory text, that it is essential to “Value the nature of science, continuing the development of scientific methodology in its different stages” (Portuguese Government/Ministry of 2018a p3, 2018b p3; 2018c p3; 2018d p4). To overcome this issue, we proposed a guideline suggesting that such statements should be coded as belonging to category 5 (NoS) without detailing the subcategory, therefore they should be coded as subcategory 5.0 (Table 4).

FACE (Table 4) will provide researchers a tool supported by validity and reliability evidence to analyze different countries’ curricula. Although countries differ significantly in several aspects (e.g., educational policies, curricula, teachers’ education, school textbooks), having a tool supported by evidence to assess coverage of evolutionary key concepts by school curricula could help researchers get a clearer picture for each country. When comparing, for example, the public’s acceptance of evolution in different countries (Miller et al. 2006), having an idea about the education that those people have received based on their official school curricula can help to understand the impact of the curricula in public’s evolution literacy and acceptance and inform appropriate decisions on educational policies to increase these.

### Study limitations and suggestions for further research

We should highlight that although important evolution learning goals may be addressed in Geography, Geology, or History, the learning goals of these disciplinary fields were only analyzed if these were taught in the same discipline that also addressed Biology learning goals. Furthermore, in our analysis we did not cover the curricula of the school years during which evolution is studied as a major topic or in more depth (in Portugal this is explored in the 11th grade). This may explain why some of FACE’s subcategories were not identified in any of the examined curricula (e.g. Rates of evolution vary, Evolution is often defined as a change in allele frequencies within a population, Genetic drift acts on the variation that exists in a population etc.). This did not surprise us because UECF suggests that some of these subcategories should be explored by older students. It is possible that additional problems could arise when using the FACE in upper educational levels. Given this, future work should expand the study of FACE’s usefulness in evaluating higher grades’ curricula.

FACE (Table 4) could also be used to assess whether the evolutionary concepts are presented in other school subjects’ curricula, besides biology, and could also be used to assess their presence in a continuity or in a fragmented fashion. The analysis of Scheuch and Rachbauer (2019) found a fragmentation of evolutionary concepts in Austrian school textbooks, and Nehm et al. (2009a, b) consider fragmentation as a major possible source of misconceptions as it gives students a fragmentary picture of evolution (Sanders and Makotsa 2016). Of course, we should not underestimate the role of a good teacher in overcoming any obstacles posed by the curriculum. Therefore, besides analyses of countries’ curricula further studies focusing on teachers, students, textbooks and teaching practices are needed to improve our understanding about how evolution is taught in each country.

## Data Availability

The datasets generated during the current study are not publicly available due to the fact that they consist part of a wider project that will present the results of a comparative analysis of European countries’ school curricula regarding evolution but are available from the corresponding author on reasonable request. Official papers referring to each country’s school curriculum that provided the original data used in this paper are the following: *Greek educational system* Government’s Gazette Vol. B, No. 304/13–03-03 [in Greek] ΑΔΑ: 6ΥΧΙ4653ΠΣ-ΧΨΕ (https://www.alfavita.gr/sites/default/files/attachments/didaktea_ili.pdf). *Portuguese educational system*. Portuguese Assembly of Republic (2009). Law n.º 85/2009, from 27 of August. https://dre.pt/application/conteudo/488826. Portuguese Government/Decree-Law n.º 55/2018, from 6 july. https://dre.pt/application/conteudo/115652962. Portuguese Government/Ministry of education, Education (2018a, b, c, d, e, f, g, h, i). *Italian educational system*. Indicazioni nazionali per il curricolo della scuola dell’infanzia e del primo ciclo d’istruzione (D.M. 254 del 16 novembre 2012): http://www.indicazioninazionali.it/2018/08/26/indicazioni-2012/. Indicazioni nazionali riguardanti gli obiettivi specifici di apprendimento concernenti le attività e gli insegnamenti compresi nei piani degli studi previsti per i percorsi liceali (D.M. n 211 del 7/10/2010); Regolamento recante norme concernenti il riordino degli istituti tecnici ai sensi dell’articolo 64, comma 4, del decreto legge 25 giugno 2008, n. 112, convertito dalla legge 6 agosto 2008, n. 133; Regolamento recante norme concernenti il riordino degli istituti professionali ai sensi dell’articolo 64, comma 4, del decreto legge 25 giugno 2008, n. 112, convertito dalla legge 6 agosto 2008, n. 133: http://archivio.pubblica.istruzione.it/riforma_superiori/nuovesuperiori/index.html*Slovenian educational system* Eurydice (2019). Slovenia overview, https://eacea.ec.europa.eu/national-policies/eurydice/content/slovenia_en. Gov.si. Republic of Slovenia (2020). Programi in učni načrti v osnovni šoli [Programs and syllabuses in compulsory basic school]. Retrieved from https://www.gov.si/teme/programi-in-ucni-nacrti-v-osnovni-soli/.

## Appendix A

See Table 5.

**Table 5:** Threshold and key concepts of evolution by natural selection (Tibel and Harms, 2017) and examples of how these are addressed in FACE

Type of Concept	Concept	Examples of how these concepts are addressed in FACE (subcategories are identified by their codes and description; guidelines provided when needed)
Threshold concept	Temporal scale	Processes taking place at very long temporal scales: 1.1 Life has been on Earth for a long time 1.3 Large scale environmental changes (caused by geological, geophysical, astronomical factors) and biological evolution are linked 2.3 The fossil record provides evidence for evolution Processes taking place at our species temporal scale: 1.4 Anthropogenic environmental changes and biological evolution are linked 2.6 Artificial selection provides evidence for evolution Processes taking place in a generation time scale: 3.3 Living things have offspring that inherit many traits from their parents but are not exactly identical to their parents Processes taking place at diverse time scale: 1.5 Many life forms that once existed have gone extinct 1.7 Life forms/species/ change through time
	Spatial scale	Large worldwide scale 1.3 Large scale environmental changes (caused by geological, geophysical, astronomical factors) and biological evolution are linked 2.4 The geographic distribution of extant species provides evidence for evolution Ecosystem/population scale 2.6 Organisms’ features, when analysed in relation to their environment provide evidence for evolution 3.5 Natural selection acts on the variation that exists in a population Individuals’ scale 3.6 Inherited characteristics affect the likelihood of an organism’s survival and reproduction Cell/molecular scales 3.3 Living things have offspring that inherit many traits from their parents but are not exactly identical to their parents (includes the following guideline: recombination and mutations in reproductive cells result in new heritable traits and are sources of diversity)
	Probability	3.6 Inherited characteristics affect the likelihood of an organism’s survival and reproduction 3.7 Sexual selection occurs when selection acts on characteristics that affect the probability of individuals to mate
	Randomness	3.3.- Living things have offspring that inherit many traits from their parents but are not exactly identical to their parents (guideline: A learning goal considered to represent subcategory 3.3 may ask students to understand that recombination and mutations are random processes) 3.8 Genetic drift acts on the variation that exists in a population
Key concepts of natural selection: Principle Variation	Origin of variation	3.3 Living things have offspring that inherit many traits from their parents but are not exactly identical to their parents. (guideline: A learning goal considered to represent subcategory 3.3 may ask students to understand that recombination and mutations in reproductive cells result in new heritable traits and are sources of diversity)
	Individual variation	3.2 There is variation within a population 3.5. Natural selection acts on the variation that exists in a population 3.8 Genetic drift acts on the variation that exists in a population
	Differential fitness	3.6 Inherited characteristics affect the likelihood of an organism’s survival and reproduction 3.9 Fitness is reproductive success — the number of viable offspring produced by an individual in comparison to other individuals in a population/species
Key concepts of natural selection: Principle Inheritance	Heritable traits	3.6 Inherited characteristics affect the likelihood of an organism’s survival and reproduction 3.3 Living things have offspring that inherit many traits from their parents but are not exactly identical to their parents
	Reproduction	3.7 Sexual selection occurs when selection acts on characteristics that affect the probability of individuals to mate 3.9 Fitness is reproductive success — the number of viable offspring produced by an individual in comparison to other individuals in a population/species
Key concepts of natural selection: Principle Selection	Selection pressure	3.6 Inherited characteristics affect the likelihood of an organism’s survival and reproduction (Guideline: A learning goal considered to represent subcategory 3.6 may ask students to understand that (…) advantageous traits depend on the environment and selective pressures it imposes)
	Differential survival and reproduction	3.6 Inherited characteristics affect the likelihood of an organism’s survival and reproduction
	Change in population	3.1 Evolution is often defined as a change in allele frequencies within a population
	Speciation	3.11 Speciation results from the splitting of one ancestral lineage into two or more descendant lineages

## Appendix B

### Description of each country’s school system and the analysed documents

#### Greece

The Greek educational system consists of preschool education (mandatory), primary education (6 years), secondary education (6 years, from which the 3 of them are mandatory—lower secondary education—and the rest optional and divided to Professional and General studies, which both can lead to exams for tertiary education) and tertiary education (the years depend on the type of studies). The Greek curricula are official documents and teachers have to follow them—only a very small deviation is allowed—and there is only one school textbook for each subject, which is available for free to students of public schools and also can be publicly accessed on the internet. The curricula that are valid today in Greece were published 17 years ago and are only being slightly modified from time to time (Government’s Gazette Vol. B, No. 304/13–03-03; ΑΔΑ: 6ΥΧΙ4653ΠΣ-ΧΨΕ). Biology, as a separate subject, exists only in secondary education, whereas in primary Education there are more general courses and teachers with no specialization in science teach these courses. Preschool students are considered to get in touch with nature and their environment and students of primary education get in touch with some biological concepts during their first 4 grades in a course called “Studying the Environment'' (referring to manmade and natural environment). During their last two years of primary education a course called Science dedicates about 1/3 of its content to Biology. However, from 2018 an official paper that was administered to schools asked teachers not to teach most of these biological concepts. As a result, Greek students who will graduate compulsory education will have studied biology mainly during their 3 last years and only for 1 h/week.

#### Portugal

In Portugal, compulsory education starts at the age of 6 years old, prior to which pre-school education is available in both private and public schools, but attendance is not mandatory (Portuguese Republic Assembly, 2009). Compulsory education extends for 12 years which include basic education and secondary education. In basic education, the subjects are common to all students (until 9th grade). In secondary education, students can attend either science or humanities courses, which have common and specific subjects for each branch, or they can attend professional courses (Portuguese Government/Decree-Law 55/2018). Basic Education comprises three cycles. The first cycle is attended by students from 6–10 years-old and extends from the 1st to the 4th grade. During this cycle a single teacher is responsible to teach several subjects that include portuguese, mathematics, study of the environment, artistic education, physical education and citizenship and development. 1st cycle students also have to attend English lessons (Diário da Républica Eletrónico/Decree-Law 55/2018). During this cycle, biology education is addressed in the study of the environment learning goals, which aim to develop content knowledge and skills in biology, geology, physics, chemistry, geography, history and technology (Portuguese Government/Ministry of Education, 2018a, b, c, d). In the second cycle, from 5 to 6th grade (11-12y students), and third cycle, from 7 to 9th grade (13-15y students), each disciplinary field is taught by specialized teachers in that field. Biology is taught in the discipline of Natural Sciences, together with Geology. For all these cycles and disciplinary fields, the learning goals in terms of content knowledge and skills are mostly determined by the national official programs, with the school boards being allowed to determine up to 25% of the annual standards. The essential learning goals documents detail the content learning and skills that should be developed by all students during basic education. For that reason, in the present study we analysed the essential learning goals of study of the environment (from the 1st to the 4th grade; Portuguese Government/Ministry of 2018a to d) and natural sciences (from the 5th to the 9th grade Portuguese Government/Ministry of Education, 2018e to i).

#### Italy

The Italian School System is organized in two cycles. The First Instruction Cycle is composed of Kindergarten, (3 to 6 years, non-compulsory education), Primary School (1st to 5th grade, from 6 to 11 years of age, compulsory education) and Lower Secondary School (6th to 8th grade, from 11 to 14 years of age, compulsory education). The First Instruction Cycle offers the same curriculum to all students. The Second Instruction Cycle is composed of Upper Secondary School (9th to 13th grade). It is compulsory until 16 years of age (9th and 10th grades) and is divided into different school types: *Liceo*, Technical and Vocational High Schools, each one with further specializations. *Liceo* High School is a general high school preparing for university, Technical High School prepares for employment but also gives access to university, Vocational High School, is more focused on practical subjects and work experience, but gives access to university too. In Kindergarten and in Primary School, a teacher (generally without a specific academic background) can teach all subject areas, including Biology, present in both segments with different names. In Lower Secondary School, science and mathematics are taught by the same teacher, usually with a biology or a mathematics degree. Science teaching time is officially 3 h per week. In *Liceo* High School, Biology is taught within Natural Sciences (2–3 h per week) by a teacher with a degree in biology/natural sciences or another related subject. In Technical and Vocational High Schools, Biology is taught as in *Liceo*, (within Natural Sciences, 2 h per week) but only in the first two grades, with the exception of some specializations (e.g. agriculture). School curricula are nationwide and regulated at the general level by Ministry of Education guidelines. Teachers are guaranteed a good amount of liberty in teaching and in choosing among many different textbooks.

#### Slovenia

In Slovenia, pre-school education is optional. Children can enrol as early as at the age of 11 months and attend it until they start compulsory school. Nine-year compulsory school is divided into three three-year cycles (for students from 6 to 14 years old). It is mandatory, 99% public, and state-financed. The first six years can be recognised as the primary (ISCED 1) level. Grades 7–9 are internationally recognised as the lower secondary school (ISCED 2). This is followed by the upper secondary school system (ISCED 3) (from two up to five years) (Eurydice, 2019). Biology learning objectives are already included in the curriculum for pre-school education, in one of six programs named Nature. In the nine-year compulsory school biology education is included in four compulsory school subjects: Learning about the environment (1st, 2nd and 3rd grade), Science and Technology (4th and 5th grade), Science (6th and 7th grade), and Biology (8th and 9th grade). The subject “Science” dedicates about 2/3 of its content to biology. Biology education is also a part of upper secondary education in subjects of Biology, Science or Science and Society, depending on the study program. Primary school teachers are teaching all school subjects in the first three-years cycle and most of the second three-year cycle. In 5th and 6th grade specialized subject teachers gradually take over teaching subjects. These others teach the subjects Science in 6th and 7th grade and Biology in the 8th and 9th grade.

## Abbreviations

FACE: Framework for the Assessment of school Curricula regarding Evolution
FC: Fundação para a Ciência e a Tecnologia
GR: Greece
IT: Italy
K-9: Kindergarten to 9th grade
NoS: Nature of Science
NRC: National Research Council (USA)
PT: Portugal
SL: Slovenia
UECF: Understanding Evolution Conceptual Framework
UK: United Kingdom
